# Low-Intensity Pulsed Ultrasound Enhanced Adipose-Derived Stem Cell-Mediated Angiogenesis in the Treatment of Diabetic Erectile Dysfunction through the Piezo-ERK-VEGF Axis

**DOI:** 10.1155/2022/6202842

**Published:** 2022-07-29

**Authors:** Shiyun Liu, Chenyi Jiang, Jianlin Hu, Huixing Chen, Bangmin Han, Shujie Xia

**Affiliations:** ^1^Department of Urology, Shanghai General Hospital, Shanghai Jiao Tong University School of Medicine, Shanghai 200080, China; ^2^Department of Andrology, The Center for Men's Health, Urologic Medical Center, Shanghai Key Laboratory of Reproductive Medicine, Shanghai General Hospital, Shanghai Jiao Tong University School of Medicine, Shanghai 200080, China

## Abstract

**Objectives:**

Erectile dysfunction is a major comorbidity of diabetes. Stem cell transplantation is a promising method to treat diabetic erectile dysfunction. In this study, we evaluated whether low-intensity pulsed ultrasound (LIPUS) could enhance the efficacy of adipose-derived stem cells (ADSCs) and investigated the underlying molecular mechanism. *Materials and methods*. Sixty 8-week-old male Sprague–Dawley rats were randomly divided into the normal control (NC) cohort or the streptozocin-induced diabetic ED cohort, which was further subdivided into DM, ADSC, LIPUS, and ADSC+LIPUS groups. Rats in the ADSC or ADSC+LIPUS group received ADSC intracavernosal injection. Rats in the LIPUS or ADSC+LIPUS group were treated with LIPUS. The intracavernous pressure (ICP) and mean arterial pressure (MAP) were recorded at Day 28 after injection. The corpus cavernosum tissues were harvested and subjected to histologic analysis and ELISA. The effects of LIPUS on proliferation and cytokine secretion capacity of ADSCs were assessed in vitro. RNA sequencing and bioinformatic analysis were applied to predict the mechanism involved, and western blotting and ELISA were used for verification.

**Results:**

Rats in the ADSC+LIPUS group achieved significantly higher ICP and ICP/MAP ratios than those in the DM, ADSC, and LIPUS groups. In addition, the amount of cavernous endothelium and cGMP level also increased significantly in the ADSC+LIPUS group. In vitro experiments demonstrated that LIPUS promoted proliferation and cell cycle progression in ADSCs. The excretion of cytokines such as CXCL12, FGF2, and VEGF was also enhanced by LIPUS. Bioinformatic analysis based on RNA sequencing indicated that LIPUS stimulation might activate the MAPK pathway. We confirmed that LIPUS enhanced ADSC VEGF secretion through the Piezo-ERK pathway.

**Conclusion:**

LIPUS enhanced the curative effects of ADSCs on diabetic erectile dysfunction through the activation of the Piezo-ERK-VEGF pathway. ADSC transplantation combined with LIPUS could be applied as a synergistic treatment for diabetic ED.

## 1. Introduction

Erectile dysfunction (ED), which is defined as the persistent failure to achieve and/or maintain sufficient erection to attain satisfactory sexual intercourse, is common in men suffering from diabetes mellitus (DM) [1]. Not only does ED impose a heavy psychological burden on patients, but it also damages their relationship with their partners, thus vastly decreasing their quality of life. Currently, oral phosphodiesterase type 5 inhibitors (PDE5i) are the first-line treatment for ED. However, due to the pathological cavernous changes caused by diabetes, the response rates of these ED patients to PDE5is are far from satisfactory and call for more potent treatments [2].

Adipose-derived stem cells (ADSCs) are mesenchymal stem cells. Accumulating evidence shows that ADSCs have multilineage differentiation potential, immunomodulatory effects, and angiogenesis-promoting ability [3]. Further studies have demonstrated that ADSCs could improve erectile function in different ED rat models [4–6]. Meanwhile, the large abundance of ADSCs in easily accessible subcutaneous adipose tissues makes ADSCs an attractive solution for medical usage [7]. Nonetheless, hypocellular activity after injection and a low retention rate in the cavernosum still restrain the practical application of ADSC-based therapy [6].

Low-intensity pulsed ultrasound (LIPUS) is a kind of special ultrasound that transmits as a pulsed wave and carries less energy (usually less than 3 W/cm^2^) than traditional ultrasound. It is generally delivered at a frequency ranging from 1 to 3 MHz and pulsed at 1 kHz. To date, the efficiency of LIPUS on ED has been demonstrated in both preclinical and clinical studies [8–10]. Increasing evidence suggests that LIPUS-mediated stem cell activation plays a pivotal role in tissue regeneration [11–13]. Therefore, it is worthwhile to investigate whether ADSC transplantation supplemented with LIPUS treatment would result in a better clinical outcome.

In the present study, we established diabetic ED rat models with streptozocin. Erectile function improvements were assessed with ICP measurement. The histological changes were evaluated via immunofluorescence staining and ELISA. Based on in vitro experiments, we researched the effects of LIPUS on the proliferation and secretion ability of ADSCs. In addition, the molecular mechanism behind LIPUS-mediated ADSC activation was also revealed.

## 2. Materials and Methods

### 2.1. ADSC Culture and Identification

Sprague–Dawley rat adipose-derived stem cells (ADSCs) were obtained from Cyagen (Guangzhou, China). The cells were cultured in ADSC complete medium (Cyagen, China). The media were replaced every 2 days, and at passages 3-4, the cells were used for experiments. Osteogenic induction and adipogenic induction assays were employed for the identification of stem cells. For osteogenic differentiation, approximately 2 × 10^4^ cells per well were seeded onto a 6-well plate. After the ADSCs reached 70% confluence, the media were replaced with osteogenic differentiation media (Cyagen, China). The media were replaced every 3 days. After a 2-week osteoinduction, the calcium nodules were stained with alizarin red S. For adipogenic differentiation assays, ADSCs were seeded at a density of 2 × 10^4^ cells per well in a 6-well plate. Cells were incubated for 21 days in adipogenesis induction media (Cyagen, China), which was replaced every 3 days. The lipid droplets were stained with oil red O solution (Cyagen, China). Surface marker identification was performed using a Rat ADSC Analysis Kit (Cyagen, China) according to the manufacturer's recommended protocol.

### 2.2. Animal Model Establishment and ADSC Transplantation

Sixty 8-week-old male Sprague–Dawley rats were purchased from the animal center of Shanghai General Hospital. After fasting for 12 hours, 10 randomly selected rats were injected intraperitoneally with vehicle (0.1 mol/L citrate solution, pH 4.5, Regal, Shanghai, China) and were enrolled in the normal control (NC) group. The remainder was injected intraperitoneally with a freshly prepared 50 mg/kg streptozocin (STZ) citrate solution (Sigma–Aldrich, St. Louis, MO). The tail vein blood glucose was monitored 72 hours after the intraperitoneal injection. Only rats with fasting blood glucose over 16.7 mmol/L were regarded as diabetic and screened for further studies. Apomorphine (100 *μ*g/kg, Macklin, Shanghai, China) was used for screening diabetic ED rats according to a previous study [14]. Forty successfully established diabetic ED rat models were randomly divided into the DM control (DM) group, ADSC transplantation (ADSC) group, LIPUS treatment (LIPUS) group, and ADSC transplantation plus LIPUS treatment (ADSC+LIPUS) group.

Rats were anesthetized with Zoletil 50 (50 mg/kg, Virbac, France) intramuscular injection after 4 weeks of STZ induction. Rats in the NC group, DM group, and LIPUS group received an intracavernous injection of 200 *μ*l PBS. A total of 1 × 10^6^ ADSCs suspended in 200 *μ*l PBS were injected into the cavernosum of rats in the ADSC group and ADSC+LIPUS group. CM-Dil (YEASEN, Shanghai, China)-labeled ADSC intracavernous engraftment was implemented to track the fate of transplanted stem cells. Briefly, ADSCs were incubated with CM-Dil working buffer at room temperature for 5 minutes and then at 4°C for another 15 minutes. Then, the cells were rinsed with PBS twice and collected for transplantation. Approximately 1 × 10^6^ CM-Dil-labeled ADSCs were engrafted as described before. One, 3, and 5 days later, the rats were sacrificed, penis tissues were harvested, and the nuclei were stained with DAPI. Images were captured with fluorescence microscopy. All animal experimental protocols were approved by the Medical Ethics Committee of the Shanghai General Hospital.

### 2.3. LIPUS Treatment

Immediately after ADSC transplantation, rats in the LIPUS group and ADSC+LIPUS group were treated with 200 mW/cm^2^ ultrasound for 5 minutes, each time delivered by a low-intensity pulsed ultrasound therapeutic machine (WanBeiLi, Beijing, China). The frequency of ultrasound was set at 1.7 MHz, and the pulse interval ratio was adjusted to 1 : 4 (200 *μ*s:800 *μ*s). Rats were treated with LIPUS 3 times per week for a total of 2 weeks.

LIPUS treatment was applied to cultured ADSCs in vitro. The cell media were refreshed ahead of ultrasound exposure. A thin layer of ultrasound gel was applied to the LIPUS transducer before it was tightly appressed to the bottom of the culture plate. The cells were then exposed to ultrasound stimulation under the following conditions: a frequency of 1.7 MHz, a pulse duty cycle of 1 : 4 (200 *μ*s:800 *μ*s), different energy intensities (100 mW/cm^2^, 200 mW/cm^2^, 300 mW/cm^2^), and an exposure time of 5 minutes.

### 2.4. Erectile Function Evaluation

Two weeks after the final LIPUS treatment, intracavernous pressure (ICP) and mean arterial blood pressure (MAP) were detected to evaluate erectile function as previously described [8]. The major pelvic ganglion (MPG) and cavernous nerve (CN) were exposed via lower abdomen midline laparotomy. The cavernosum was cannulated with a heparinized (250 U/ml) 23-gauge butterfly needle connected to a multichannel physiological signal recorder (MP160, Biopac Systems, Goleta, CA). A stainless electrode hook was utilized to stimulate CNs. The stimulus parameters were 5 V, 20 Hz, pulse width of 5 msec, and a duration of 50 seconds. The maximum ICP increases of three stimulations were recorded for statistical analysis for each rat. MAP was recorded using a 23-gauge needle inserted into the right carotid artery. The ICP/MAP ratio was calculated for each rat to eliminate individual differences, and the mean ICP/MAP ratio was then used for statistical analysis.

### 2.5. Immunohistology

Rats were sacrificed after erectile function evaluation, and their penises were harvested, fixed, and sliced into 5-*μ*m sections for immunofluorescence assays. The immunofluorescent staining procedures were described in [15]. Primary antibodies against eNOS (Servicebio, Wuhan, China) and Von Willebrand Factor (Servicebio, Wuhan, China) were used. 4′,6-diamidino-2-phenylindole (DAPI) was applied to stain nuclei.

### 2.6. Cell Viability Assay

ADSCs were seeded into 96-well plates at a density of 1 × 10^4^ cells per well. All adherent cells were treated with LIPUS as described before. After incubation at 37°C for 24 hours, ADSCs in each well were incubated with 10 *μ*l CCK8 solution for 2 hours. The absorbance was detected at 450 nm using a microplate reader.

### 2.7. Colony Formation Assay

Approximately 500 ADSC cells were seeded into each well of 6-well plates. LIPUS treatment was carried out 3 times per week. After 14 days of incubation, clones were fixed and stained with crystal violet.

### 2.8. EdU Assay

The proliferation ability of ADSCs was measured with a 5-ethynyl-20-deoxyuridine (EdU) assay kit (Beyotime, Shanghai, China). In brief, ADSCs were seeded into confocal plates at a density of 5 × 10^5^ cells per well. Then, 10 *μ*M EdU buffer was added to the culture medium. Cells in the LIPUS group were treated with LIPUS as described before. After incubation at 37°C in the dark for 4 hours, ADSCs were rinsed with PBS twice, fixed with 4% paraformaldehyde for 15 minutes, and permeabilized with 0.3% Triton X-100 for 10 minutes. Cell nuclei were stained with Hoechst. Images were captured with a Leica TCS SP8 confocal microscope.

### 2.9. Cell Cycle Assay

Twenty-four hours after LIPUS stimulation, ADSCs were harvested and fixed overnight in precooled 70% ethanol. A cell cycle analysis kit (Beyotime, Shanghai, China) was utilized to analyze the cell cycle of ADSCs in either the control group or the LIPUS group according to the manufacturer's instructions. Cells were stained with PI solution and detected with the FL2 channel on a BD Accuri C6 flow cytometry machine.

### 2.10. Quantitative Real-Time PCR (qRT–PCR)

TRIzol reagent (Life Technologies, CA, USA) was used to extract total RNA from ADSCs 2 hours after LIPUS stimulation. A PrimeScript RT reagent kit (Takara, Otsu, Japan) was used to generate cDNA. Quantitative real-time PCR using a TB Green Premix Ex Taq II kit (Takara, Otsu, Japan) was carried out on a QuantStudio™ 6 Flex Real-Time PCR system. The 2 -*ΔΔ*Ct method was employed to calculate the gene expression compared to GAPDH as the internal control. The primers used in this study are listed in Supplementary Table 1.

### 2.11. Enzyme-Linked Immunosorbent Assay (ELISA)

Approximately 50 mg of cavernous tissues were homogenized in tissue lysis buffer special for nitric oxide assay (Beyotime, Shanghai, China) and centrifuged at 12000 × g for 5 minutes. The supernatant was collected for the BCA assay (Beyotime, Shanghai, China) to measure the protein concentration of each sample. The cavernous cGMP level was determined using a commercial ELISA kit (Jiangsu Meibiao Biotechnology, Jiangsu, China).

Two hours after LIPUS treatment, the supernatant of ADSCs was collected for ELISAs. The samples were analyzed using CXCL12, FGF2, VEGF, NGF, HGF, and IGF1 commercial ELISA kits obtained from Jiangsu Meibiao Biotechnology (Jiangsu, China) according to the manufacturer's protocol. The absorbance was measured using a microplate reader.

### 2.12. RNA Sequencing and Bioinformatic Analysis

Two hours after the initiation of LIPUS treatment, the total RNA from ADSCs in either the control group or the LIPUS group was used for RNA sequencing, performed by Genergy Biotechnology Inc. (Shanghai, China). Differentially expressed genes (DEGs) were identified with the DESeq2 R package based on the criteria of |log2FC| ≥ 1 and *p* value <0.05 [16]. The top 30 DEGs were presented using the pheatmap R package [17]. Gene Ontology (GO) and Kyoto Encyclopedia of Genes and Genomes (KEGG) analyses were performed using the online web tool DAVID [18, 19]. The STRING database (Version: 11.5) was used to generate protein–protein interaction (PPI) networks [20]. The Cytoscape plugin cytoHubba was used to identify the top 12 hub genes.

### 2.13. Western Blotting

ADSCs were pretreated with PD98059 (HY-12028, 80 *μ*M, MedChemExpress), GsMTx4 (HY-P1410A, 5 *μ*M, MedChemExpress), and Yoda1 (HY-18723, 30 *μ*M, MedChemExpress) for 1 hour as requested, and the cells were harvested 2 hours after LIPUS stimulation without specification. ADSCs were lysed on ice with RIPA buffer (Beyotime, Shanghai, China) containing the protease inhibitor PMSF (Beyotime, Shanghai, China) and Phosphatase Inhibitor Cocktail (YEASEN, Shanghai, China). The protein concentrations of cell lysates were measured using a BCA protein assay kit (Beyotime, Shanghai, China). Protein samples (15 *μ*g) were separated on 10% SDS-polyacrylamide gels and transferred to polyvinylidene fluoride membranes. The membranes were then blocked with 10% bovine serum albumin (BSA) for 1 hour at room temperature and incubated with primary antibodies at 4°C overnight. The primary antibodies used in this study were ERK (ab184699, 1 : 10000, Abcam, USA), p-ERK (ab76299, 1 : 5000, Abcam, USA), VEGFA (ab214424, 1 : 1000, Abcam, USA), and GAPDH (ab181602, 1 : 10000, Abcam, USA). After being rinsed with TBST, the membranes were hybridized with secondary antibodies and reacted with ECL solution (NCM Biotech Co., Ltd, Suzhou, China). The chemiluminescent images were taken, and the density of each protein band was determined using ImageJ software.

### 2.14. Statistical Analysis

The results were analyzed using GraphPad Prism 7.00 (GraphPad Software Inc., La Jolla, CA, USA) and are presented as the mean ± standard deviation. Two-tailed Student's *t* test was performed for statistical analysis between two groups. One-way ANOVA followed by Tukey's multiple comparison test was used for comparisons among groups. A *p* value <0.05 was considered statistically significant. All experiments were independently implemented at least three times.

## 3. Results

### 3.1. Identification of ADSCs and Biosafety Assessment of LIPUS

**Figure 1:**
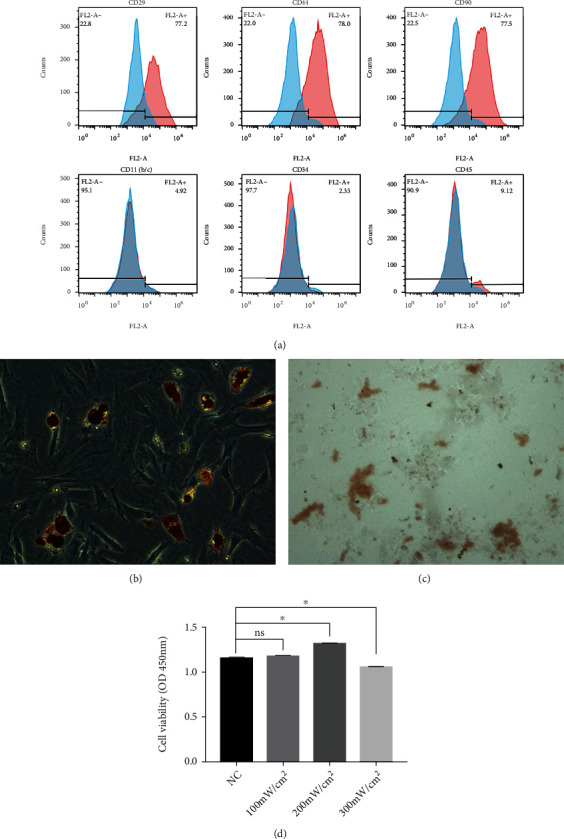
Characterization of ADSCs. (a) ADSCs expressed MSC markers (CD29, CD44, and CD90) but were negative for CD11b/c, CD34, and CD45. (b) After adipogenic induction, the accumulation of lipid droplets in ADSCs was stained with Oil Red O. (c) After osteogenic induction, the accumulation of mineralized nodes in ADSCs was stained with alizarin red S. D The cell viability of ADSCs without or with 100 mW/cm^2^, 200 mW/cm^2^, and 300 mW/cm^2^ intensities of LIPUS treatment. Data are presented as the mean ± standard deviation. ns *p* > 0.05 compared with the NC group. ∗*p* < 0.05 when comparing the NC group.

Flow cytometry was implemented to investigate the cell membrane antigens of ADSCs. The results demonstrated that ADSCs were positive for MSC markers (CD29, CD44, and CD90) but negative for CD11b/c, CD34, and CD45 (Figure 1(a)). The multilineage differentiation potential of ADSCs was also examined. The accumulation of lipid droplets in adipogenic ADSC cytoplasm was shown by Oil Red O staining (Figure 1(b)). Alizarin red staining detected mineralized nodes in osteogenic ADSCs (Figure 1(c)). LIPUS of different intensities was applied to ADSCs to evaluate its biosafety. The results of CCK8 demonstrated that 100 mW/cm^2^ LIPUS had little effect on the proliferation of ADSCs, while LIPUS with an intensity of 300 mW/cm^2^ significantly inhibited cell proliferation. However, LIPUS stimulation at 200 mW/cm^2^ promoted a significant increase in ADSC proliferation (Figure 1(d)). Therefore, LIPUS at 200 mW/cm^2^ was safe and effective for the stimulation of ADSCs.

### 3.2. ADSC Therapy Combined with LIPUS Improves Erectile Function in Diabetic ED Rat Models

**Figure 2:**
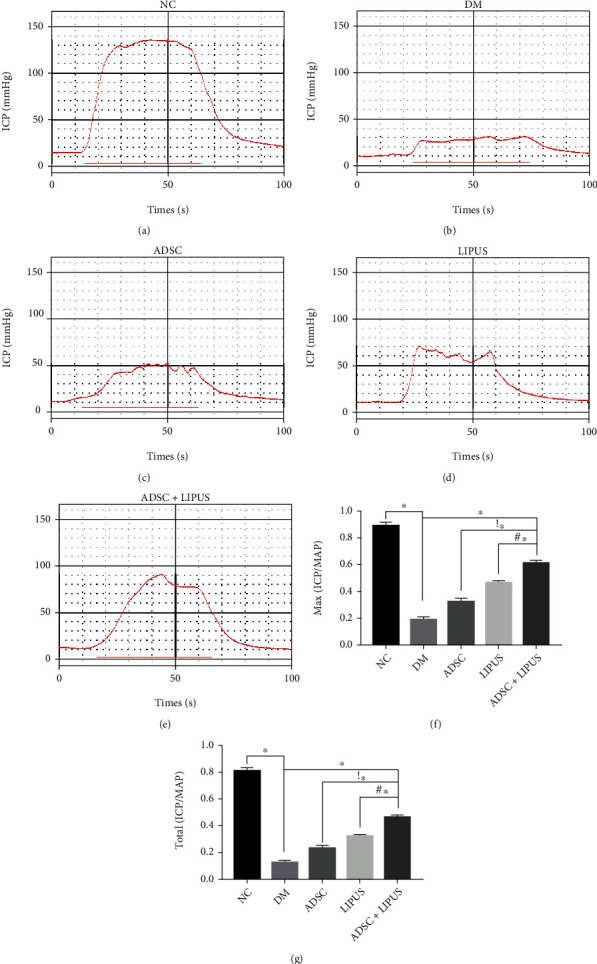
Erectile function at 4 weeks after the initiation of treatment. (a–e) Representative intracavernous pressure (ICP) responses to cavernous nerve (CN) electrostimulation from the NC, DM, ADSC, LIPUS, and ADSC+LIPUS groups. The red bar denotes the 50-second CN electrical stimulation. (f, g) The ratios of maximal ICP to mean arterial pressure (MAP) and total ICP to total MAP were recorded. Data are presented as the mean ± standard deviation. ∗*p* < 0.05 when comparing the NC group. !∗*p* < 0.05 when comparing the ADSC group. #∗*p* < 0.05 when comparing the LIPUS group.

A streptozotocin-induced diabetic ED model was established in rats. After STZ injection, rats with blood glucose levels ≥16.7 mmol/L were screened out for further studies. Compared to the NC group (0.89 ± 0.03, 0.81 ± 0.02), the maximal ICP/MAP ratios and total ICP/MAP ratios significantly decreased in the DM group (0.19 ± 0.02, 0.12 ± 0.01), which indicated successful establishment of the diabetic ED model (Figures 2(a) and 2(b)). Intracavernous injection of ADSCs (0.32 ± 0.03, 0.23 ± 0.01), LIPUS treatment (0.46 ± 0.01, 0.32 ± 0.02), and intracavernous injection of ADSCs combined with LIPUS treatment (0.61 ± 0.02, 0.46 ± 0.03) improved erectile function to varying degrees, as reflected by the significantly elevated maximal ICP/MAP ratios and total ICP/MAP ratios (Figures 2(c)–2(e)). The significant increase in maximal ICP/MAP ratios and total ICP/MAP ratios in the ADSC+LIPUS group compared to either the ADSC or LIPUS group suggested that synergistic therapy achieved the best recovery of erectile function in diabetic rats (Figures 2(f) and 2(g)). Moreover, we monitored the fate of transplanted stem cells using CM-Dil-labeled ADSCs. Both the ADSC and ADSC+LIPUS groups' cavernous samples contained a significant number of stem cells one day after transplantation. Three days later, more stem cells remained in the ADSC+LIPUS group. On the fifth day after transplantation, more stem cells were detected in the ADSC+LIPUS group, while few cells were present in the ADSC group. These findings indicated that LIPUS stimulation might prolong the retention of ADSCs in the corpus cavernosum (Supplementary Figure 1).

### 3.3. ADSC Therapy Combined with LIPUS Improves the Cavernous Endothelium in Diabetic ED Rat Models

**Figure 3:**
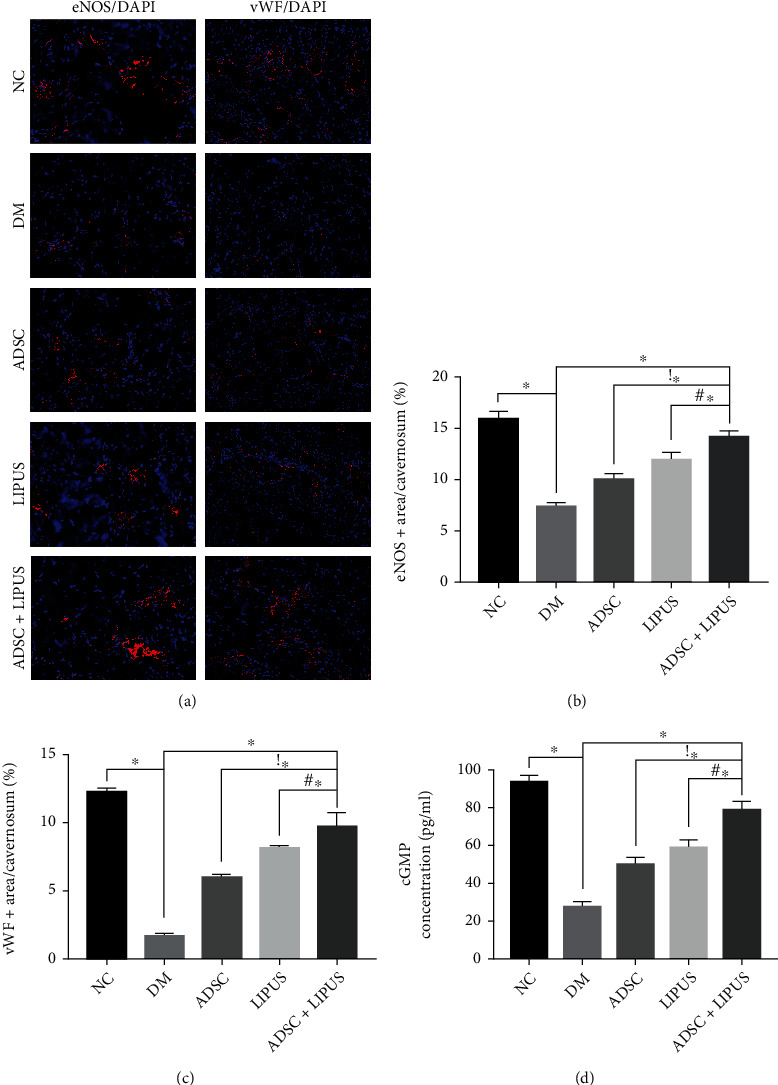
Endothelial content variations in the corpus cavernosum. (a) Representative immunofluorescence staining of eNOS (red) and vWF (red) in penile tissues from the NC, DM, ADSC, LIPUS, and ADSC+LIPUS groups. (b) Quantitative data of cavernous eNOS-positive endothelial cell content using ImageJ. (c) Quantitative data of cavernous vWF-positive endothelial cell content using ImageJ. (d) Cavernous cGMP levels in each group were assessed with ELISA. Data are presented as the mean ± standard deviation. ∗*p* < 0.05 when comparing the NC group. !∗*p* < 0.05 when comparing the ADSC group. #∗*p* < 0.05 when comparing the LIPUS group.

Since the cavernous endothelium is fundamental for normal erections, we analyzed the expression of the endothelial markers eNOS and vWF via immunofluorescence to assess changes in cavernous vascular density. The results demonstrated that eNOS- and vWF-positive areas were significantly decreased in the DM group compared to the NC group. ADSC transplantation, LIPUS treatment, and synergistic therapy significantly increased the ratio of eNOS- and vWF-positive areas (Figure 3(a)). In addition, the eNOS- and vWF-positive areas were significantly larger in the synergistic group than in either the ADSC or LIPUS group (Figures 3(b) and 3(c)). ADSC therapy combined with LIPUS had beneficial effects on the endothelial contents in the cavernosum. As the cavernous endothelium exerts physiological functions through the eNOS-NO-cGMP pathway to mediate vasodilation and contribute to erection, we detected the level of cGMP in the corpus cavernosum to evaluate its function.  Figure 3(d) shows that cGMP levels increased in the ADSC, LIPUS, and ADSC+LIPUS groups, while ADSC therapy combined with LIPUS promoted a more significant increase. To conclude, these results indicated that synergistic therapy resulted in superior cavernous vascular improvement compared with monotherapies.

### 3.4. LIPUS Therapy Enhances ADSC Proliferation

**Figure 4:**
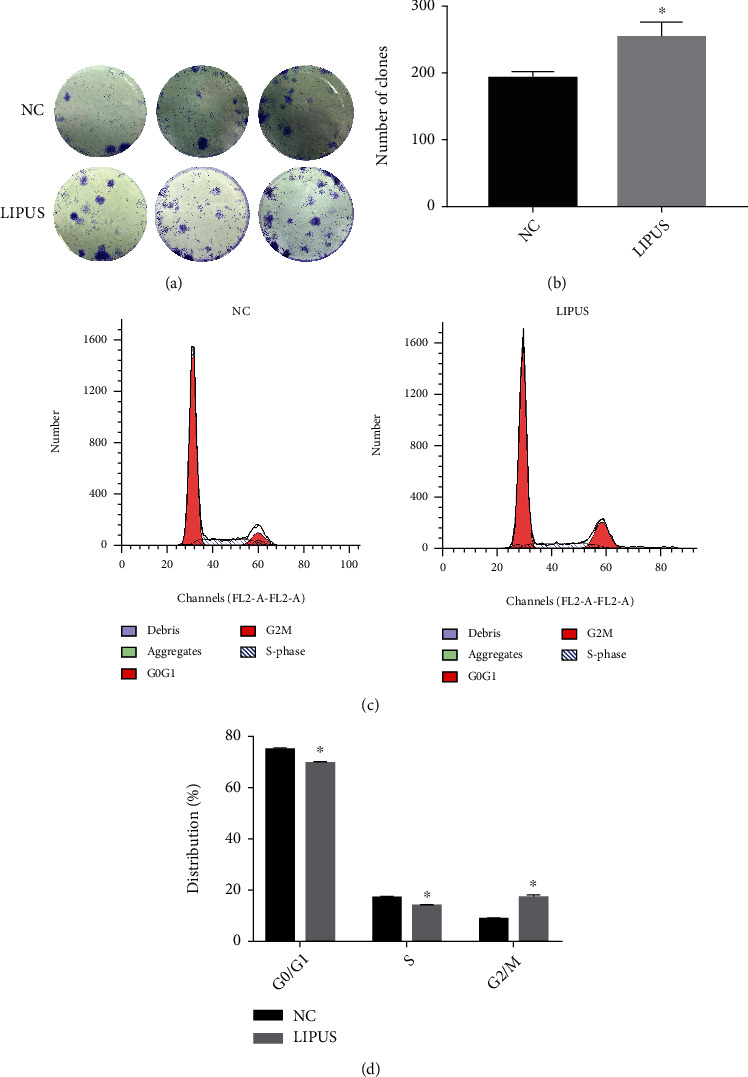
LIPUS promoted ADSC proliferation. (a), B Colony formation of ADSCs without and with LIPUS stimulation. (c), D The cell cycle distribution of ADSCs without and with LIPUS stimulation. Data are presented as the mean ± standard deviation. ∗*p* < 0.05 when comparing the NC group.

Although the results of in vivo studies were encouraging, the interaction between LIPUS and ADSCs still has not yet been discussed. Therefore, we conducted in vitro experiments to further investigate the impacts of LIPUS on ADSCs. Colony formation assay results showed that LIPUS-treated ADSCs formed significantly more colonies than those in the control group, indicating LIPUS could promote cell proliferation (Figures 4(a) and 4(b)). Moreover, cell cycle analysis demonstrated that LIPUS treatment decreased the proportion of cells in the G0/G1 and S phases but increased the number of cells in the G2 phase (Figures 4(c) and 4(d)). The results of the EdU assay also showed that the ratio of cells stained blue and red, which represents proliferating cells, was significantly greater in the LIPUS stimulation group (Supplementary Figure 2). These results suggested that LIPUS enhanced the proliferation of ADSCs through the promotion of the S-to-G2 phase transition.

### 3.5. LIPUS Therapy Promotes Cytokine Secretion by ADSCs

**Figure 5:**
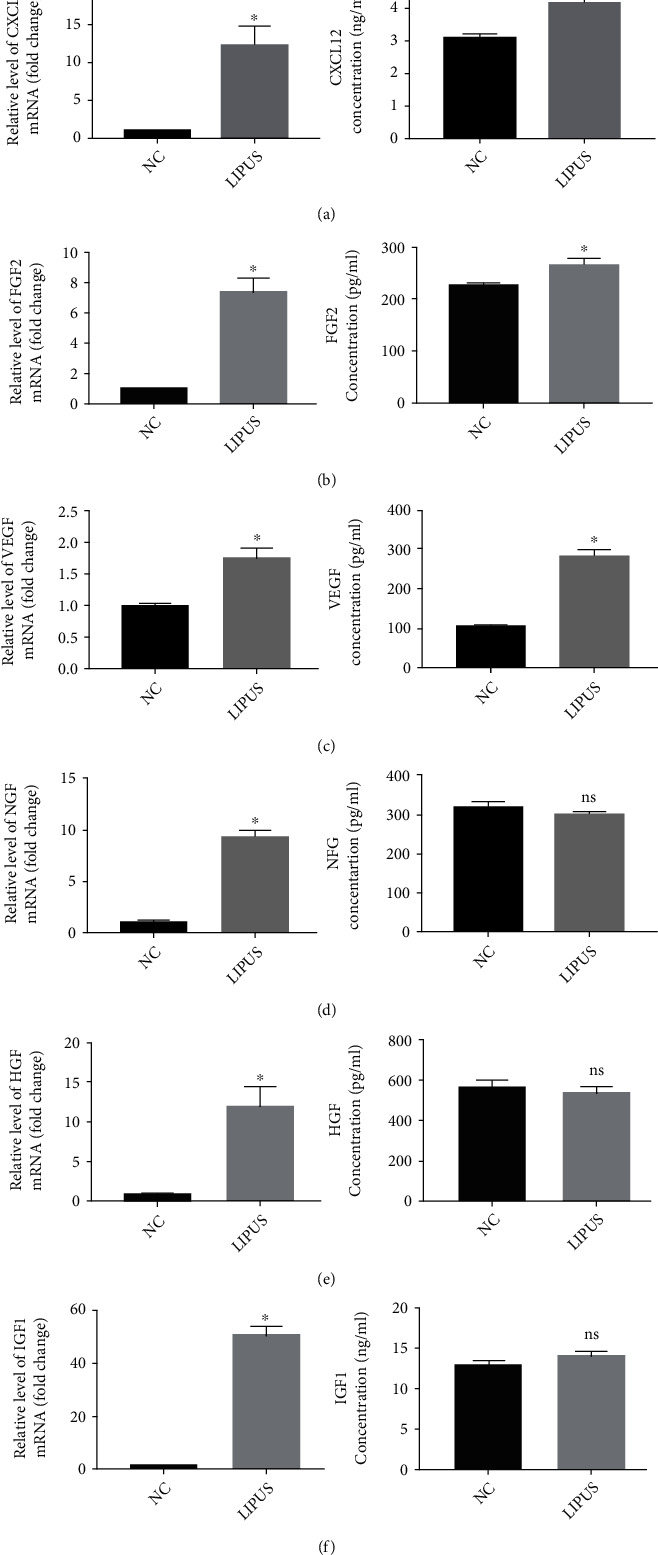
LIPUS promoted ADSCs to secrete cytokines. (-Fa–f) The CXCL12, FGF2, VEGF, NGF, HGF, and IGF1 mRNA expression levels and the concentrations of CXCL12, FGF2, VEGF, NGF, HGF, and IGF1 in the supernatant of the cell culture medium were measured using qRT–PCR and ELISA. Data are presented as the mean ± standard deviation. ns *p* > 0.05 when comparing the NC group. ∗*p* < 0.05 when comparing the NC group.

Given the pivotal role of cytokines in tissue regeneration, we further evaluated the effects of LIPUS on the paracrine function of ADSCs. qRT–PCR elucidated that LIPUS stimulation significantly upregulated the mRNA expression levels of cytokines such as CXCL12, FGF2, VEGF, NGF, HGF, and IGF1 (Figures 5(a)–5(f)). Furthermore, the concentrations of CXCL12, FGF2, and VEGF in the supernatant were augmented after LIPUS treatment, whereas no significant changes were observed in the levels of NGF, HGF, and IGF1. These data indicated that LIPUS promoted ADSCs to secrete cytokines such as CXCL12, FGF2, and VEGF.

### 3.6. Bioinformatic Analysis of LIPUS-Mediated ADSC Activation

**Figure 6:**
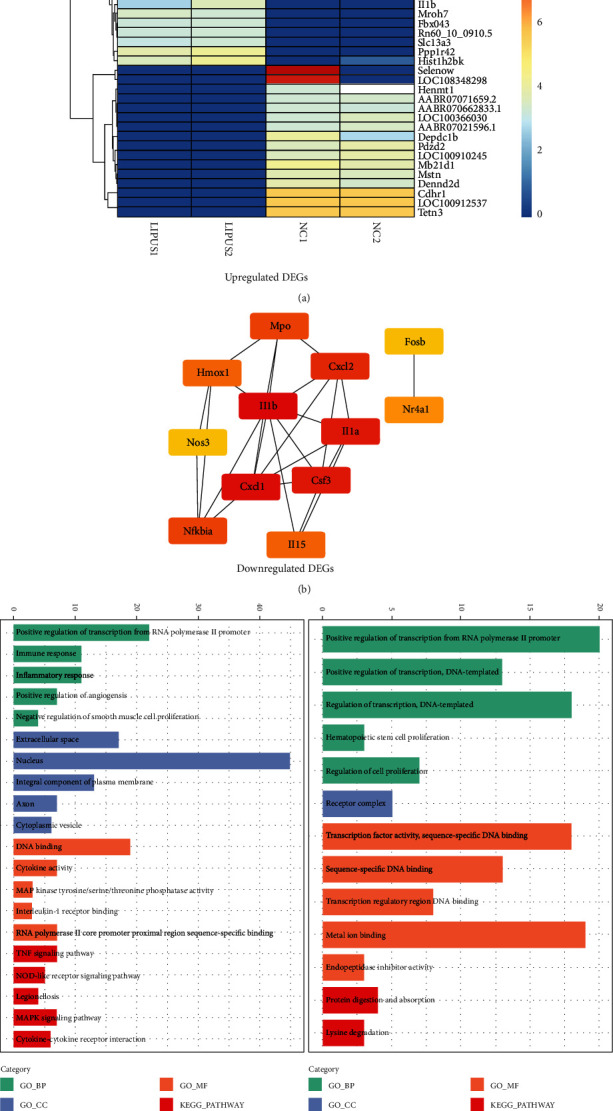
Transcriptome bioinformatic analysis of ADSCs without and with LIPUS stimulation. (a) Heatmap plot of significantly and differentially expressed genes (DEGs) of ADSCs without and with LIPUS stimulation. (b) The top 12 hub genes of ADSCs without and with LIPUS stimulation were analyzed by CytoHubba. (c) GO and KEGG analyses of upregulated DEGs and downregulated DEGs.

To explore the mechanism of LIPUS-mediated ADSC activation, RNA-seq was performed to conduct gene expression profiling analysis. Among 354 differentially expressed genes, LIPUS treatment upregulated 174 genes and downregulated 180 genes. The heat map plot presented the top 30 genes with the greatest changes in expression (Figure 6(a)). DAVID Bioinformatics Resources was utilized to analyze Gene Ontology (Figure 6(c)). The results showed that LIPUS regulated the biological processes involved in the immune response, inflammatory response, and positive regulation of angiogenesis. In terms of molecular function, cytokine activity, MAP kinase tyrosine/serine/threonine phosphatase activity, and interleukin-1 receptor binding were found to be activated. KEGG pathway analysis identified the LIPUS-mediated activation of the TNF signaling pathway, NOD-like receptor signaling pathway, MAPK signaling pathway, and cytokine–cytokine receptor interaction. However, protein digestion and absorption and lysine degradation were predicted to be inhibited. Afterward, the protein–protein interaction network was constructed using STRING database (Supplementary Figure 3). CytoHubba was further used to analyze the top 12 hub genes, including Csf3, Cxcl1, Cxcl2, Fosb, Hmox1, IL15, IL1a, IL1b, Mpo, Nfkbia, Nos3, and Nr4a1 (Figure 6(b)). Bioinformatic analysis indicated that LIPUS might trigger the activation of the MAPK pathway and angiogenesis.

### 3.7. Effects of LIPUS on the ADSC ERK-VEGF Pathway

**Figure 7:**
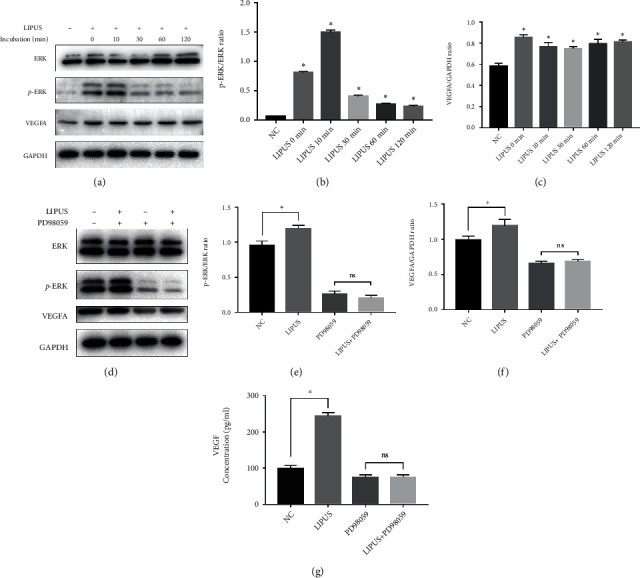
LIPUS activated the ERK-VEGFA pathway in ADSCs. (a) Western blot analysis of ERK, p-ERK, VEGFA, and GAPDH expression in ADSCs treated without or 0 minutes, 10 minutes, 30 minutes, 60 minutes, and 120 minutes after LIPUS stimulation. (b) The relative density of p-ERK compared with that of ERK. (c) The relative density of VEGF compared with that of GAPDH. (d) Western blot analysis of ERK, p-ERK, VEGFA, and GAPDH expression in ADSCs treated with LIPUS or PD98059, an ERK1/2 pathway inhibitor (ADSCs were pretreated with 80 *μ*M PD98059 for 1 hour). (e) The relative density of p-ERK compared with that of ERK. (f) The relative density of VEGF compared with that of GAPDH. (g) The concentration of VEGF in the supernatant of the cell culture medium. Data are presented as the mean ± standard deviation. ns *p* > 0.05 when comparing the PD98059 group. ∗*p* < 0.05 when comparing the NC group.

Western blotting was then used to detect the expression levels of related proteins to verify the bioinformatic results. LIPUS barely changed the expression of total ERK in ADSCs (Figure 7(a)). However, p-ERK significantly increased immediately after LIPUS stimulation but decreased at 2 hours after treatment (Figure 7(b)). Furthermore, the expression of VEGF was also elevated after the initiation of LIPUS, which was consistent with the ELISA results (Figures 4(c) and 7(c)). To further discover the relationship between the ERK pathway and VEGF expression, PD98059, an ERK1/2 pathway inhibitor, was applied (Figure 7(d)). The upregulation of p-ERK and VEGF prompted by LIPUS was reversed after the administration of PD98059 (Figures 7(e) and 7(f)). Moreover, PD98059 abolished the LIPUS-induced enhancement of VEGF secretion from ADSCs (Figure 7(g)). These results demonstrated that LIPUS activated the ERK pathway in ADSCs, which in turn provoked VEGF expression and secretion.

### 3.8. Effects of Piezo Channels on LIPUS-Mediated ERK-VEGF Pathway Activation

**Figure 8:**
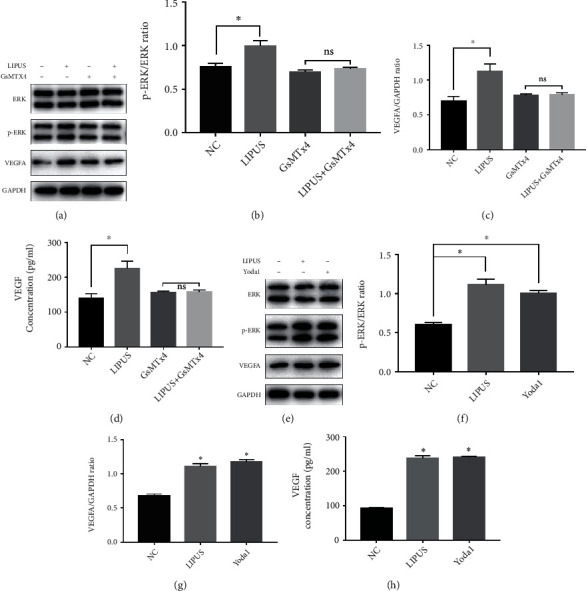
LIPUS activated the downstream pathway through the Piezo channel in ADSCs. (a) Western blot analysis of ERK, p-ERK, VEGFA, and GAPDH expression in ADSCs treated with LIPUS or GsMTX4, a selective Piezo channel inhibitor (ADSCs were pretreated with 5 *μ*M GsMTX4 for 1 hour). (b) The relative density of p-ERK compared with that of ERK. (c) The relative density of VEGF compared with that of GAPDH. (d) The concentration of VEGF in the supernatant of the cell culture medium. (e) Western blot analysis of ERK, p-ERK, VEGFA, and GAPDH expression in ADSCs treated with LIPUS or Yoda1, an agonist of the Piezo channel (ADSCs were pretreated with 30 *μ*M Yoda1 for 1 hour). (f) The relative density of p-ERK compared with that of ERK. (g) The relative density of VEGF compared with that of GAPDH. (h) The concentration of VEGF in the supernatant of the cell culture medium. Data are presented as the mean ± standard deviation. ns *p* > 0.05 when comparing the GsMTX4 group. ∗*p* < 0.05 when comparing the NC group.

The role of the Piezo ion channel in cellular mechanosensation has been reported in other studies [21, 22]. GsMTx4, a selective Piezo channel inhibitor, was used to test whether the Piezo channel dominated the activation of the ERK-VEGF pathway in ADSCs (Figure 8(a)). Following GsMTx4 treatment, there was no significant difference in the ratio of p-ERK to total ERK between the GsMTx4-treated group and the LIPUS plus GsMTx4-treated group (Figure 8(b)). Furthermore, the variation in VEGF protein levels was not observed in the LIPUS-treated group in the presence of GsMTx4 (Figure 8(c)). The ELISA results also confirmed that GsMTx4 prohibited ADSCs from secreting VEGF (Figure 8(d)). In contrast, after the application of Yoda1, an agonist of the Piezo channel, the expression of p-ERK and VEGF in ADSCs significantly increased (Figures 8(e)–8(g)). The secretion of VEGF was also promoted by Yoda1 (Figure 8(h)). These results suggested that the Piezo channel acts as an upstream activator of the ERK-VEGF pathway in LIPUS-mediated ADSC activation.

## 4. Discussion

Diabetes mellitus represents a common risk factor for erectile dysfunction, and the malfunction of the cavernous endothelium is one of the determining pathogenic factors in diabetic ED [2]. Due to the deteriorated function of the endothelium, the in situ production of cGMP decreases and makes it difficult for PDE5i, whose main pharmacological action is to prevent cGMP degradation, to exert therapeutic effects [23, 24]. ADSC engraftment has been reported by various studies to improve erectile function and histopathological changes in diabetic ED rat models [5, 6, 25–27]. However, the therapeutic effect of ADSC injection therapy is far from satisfactory. The high glucose and exaggerated oxidative stress microenvironment within the cavernosum severely diminished the viability of engrafted ADSCs, thus impairing their efficacy [28–30].

The results of our in vivo study showed that the ICP/MAP ratio was higher in the ADSC+LIPUS group than in the DM, ADSC, or LIPUS groups, indicating that the combination of ADSC transplantation with LIPUS stimulation was more efficient than monotherapy. Moreover, the concentrations of eNOS, vWF, and cGMP increased significantly in the ADSC+LIPUS group. This finding indicated that synergistic therapy promoted the repair of the cavernous endothelium, thus improving the erectile function of diabetic ED rats.

Similar to previous studies [31–34], we found that LIPUS treatment could promote the proliferation of ADSCs. Further cell cycle analysis demonstrated that the proportion of cells in the G2/M phase increased significantly in the LIPUS exposure group but decreased in the G0/G1 and S phases. Therefore, the enhanced cell viability after LIPUS treatment guaranteed that ADSCs exerted therapeutic effects, which contributed to the improved erectile function in the ADSC+LIPUS group.

Cytokines secreted by stem cells have been proven to play an essential role in tissue repair and regeneration [35, 36]. However, the role of LIPUS in the secretome of ADSCs remains unclear. We screened and identified the overexpression of CXCL12, FGF2, and VEGF in the LIPUS treatment group. CXCL12 (also known as stromal cell-derived factor 1 (SDF-1)) is a kind of chemokine that is well known for the recruitment of endogenous or transplanted stem cells [37, 38]. Recently, Yamaguchi et al. reported that local injection of SDF-1 augments neovascularization by recruiting endothelial progenitor cells [39]. Hardy et al. established a CXCL12-mutated mouse model and found that CXCL12 is crucial for angiogenesis and muscle regeneration [40]. In addition, numerous studies have demonstrated that FGF2 (fibroblast growth factor 2) and VEGF (vascular endothelial growth factor) are two predominant cytokines in angiogenesis [41–43]. Therefore, it is safe to speculate that LIPUS-mediated ADSC proangiogenic factor secretion is another reason for the modified cavernous endothelial microenvironment and improved erectile function.

Despite these encouraging results, the detailed mechanisms by which LIPUS regulates ADSCs remain poorly understood. With the help of bioinformatics analysis, we identified that LIPUS-provoked angiogenesis mediated by ADSCs, which was consistent with the results of in vivo and in vitro experiments. Furthermore, the activation of the MAPK pathway was predicted from the differentially expressed genes, which is consistent with studies on other kinds of stem cells [21, 44]. Hence, we investigated whether LIPUS could promote ADSC-induced angiogenesis by activating the MAPK pathway. ERK phosphorylation was initiated immediately after LIPUS treatment, peaked at 10 minutes, and remained at a high level within 2 hours. Zhang et al. also demonstrated that LIPUS treatment could trigger a transient intracellular influx of Ca2+ in dental pulp stem cells, thus activating the ERK signaling pathway and keeping it active for at least 30 minutes [21]. The increased VEGF expression and its elevated concentration in the supernatant were abolished after pretreatment with PD90859, a specific MEK inhibitor, further confirming the contribution of the ERK pathway to VEGF secretion from ADSCs.

Since LIPUS is a noninvasive mechanical therapy, it is intriguing how stem cells sense and transfer mechanical stimuli to activate the ERK signaling pathway, a biological process. Recently, researchers have discovered a family of proteins called Piezo and have proven their nonnegligible role in mechanobiological coupling [45–47]. Therefore, a Piezo ion channel activator, Yoda1, and inhibitor, GsMTX4, were implemented to survey the impacts of Piezo on the ERK-VEGF axis in ADSCs. We found that GsMTX4 inhibited LIPUS from phosphorylating ERK, which gave rise to the decreased secretion of VEGF in ADSCs. In contrast, Yoda1 exerted similar effects as LIPUS on ADSCs by promoting ERK activation and VEGF secretion. These results indicated that the LIPUS-dependent Piezo-ERK-VEGF axis might be one of the mechanisms by which ADSCs were activated.

However, some problems remain unsolved in this research. The low retention rate of engrafted stem cells in the cavernosum is another factor hindering their potency. Although we observed an elevated level of CXCL12 after LIPUS exposure, the role of LIPUS on the recruitment of ADSCs was not investigated in this study but will be fully addressed in future research, as will the survival of cells after transplantation. Inspired by the success of LIPUS therapy on ED patients [9, 10], Xia speculated that mechanobiological transduction mediated tissue-specific endogenous stem cells activation might play a pivotal role in the process [48, 49]. The presence and the activation of tissue-resident stem cells were not validated in this study but will be further studied in the future. Given that we utilized rat-derived stem cells to conduct our experiments, our results should first be crosschecked in cell lines derived from *Homo sapiens* before the clinical application of LIPUS combined with ADSC engraftment. However, the approach promises to achieve similar results from assays based on humanized cell lines, since Piezo, ERK, and VEGF are evolutionarily conserved proteins [45, 50–52]. Last but not least, we would like to confirm our findings in future population-based studies.

## 5. Conclusions

LIPUS enhanced the curative effects of ADSCs on diabetic erectile dysfunction through the activation of the Piezo-ERK-VEGF pathway. ADSC transplantation combined with LIPUS could be applied as a synergistic treatment for diabetic ED.

## Data Availability

All of the data generated during this study are included in this article, or if absent please contact the corresponding author.

## Abbreviations

DM: Diabetes mellitus
ED: Erectile dysfunction
LIPUS: Low-intensity pulsed ultrasound
ADSC: Adipose-derived stem cell
PDE5i: Phosphodiesterase type 5 inhibitors
STZ: Streptozocin
ICP: Intracavernous pressure
MPG: Major pelvic ganglion
CN: Cavernous nerve
eNOS: Endothelial nitric oxide synthase
vWF: Von Willebrand Factor
GO: Gene Ontology
KEGG: Kyoto Encyclopedia of Genes and Genomes
PPI: Protein–protein interaction network
CXCL12: C-X-C motif chemokine ligand 12
SDF-1: Stromal cell-derived Factor 1
FGF2: Fibroblast growth Factor 2
VEGF: Vascular endothelial growth factor.

## Supplementary Materials

Supplementary Materials Table 1: Primers used for qRT–PCR. Figure 1 The fate of transplanted ADSCs. No significant differences in the number of resident stem cells were observed one day after the transplantation. 3 and 5 days later, more ADSCs were retained in the cavernosum in the LIPUS treatment group. Data are presented as the mean ± standard deviation. ns p > 0.05 when comparing the ADSC group on the first day. ∗p < 0.05 when comparing the ADSC group on the third and fifth days. Figure 2 The EdU staining of ADSCs. The percentage of EdU-positive cells in the LIPUS treatment group was significantly higher than that in the NC group. Data are presented as the mean ± standard deviation. ∗p < 0.05 when comparing the NC group. Figure 3 The protein–protein interaction network of DEGs of ADSCs without and with LIPUS stimulation.

## References

[1] Salonia A., Bettocchi C., Boeri L. (2021). European Association of Urology guidelines on sexual and reproductive health-- 2021 Update: Male Sexual Dysfunction. *European Urology*.

[2] Castela Â., Costa C. (2016). Molecular mechanisms associated with diabetic endothelial-erectile dysfunction. *Nature Reviews Urology*.

[3] Nguyen Thanh L., Dam P. T. M., Nguyen H. P. (2021). Can autologous adipose-derived mesenchymal stem cell transplantation improve sexual function in people with sexual functional deficiency?. *Stem Cell Reviews and Reports*.

[4] Fandel T. M., Albersen M., Lin G. (2012). Recruitment of intracavernously injected adipose-derived stem cells to the major pelvic ganglion improves erectile function in a rat model of cavernous nerve injury. *European Urology*.

[5] Yang Q., Chen W., Zhang C. (2020). Combined transplantation of adipose tissue-derived stem cells and endothelial progenitor cells improve diabetic erectile dysfunction in a rat model. *Stem Cells International*.

[6] Xu Y., Yang Y., Zheng H. (2020). Intracavernous injection of size-specific stem cell spheroids for neurogenic erectile dysfunction: efficacy and risk versus single cells. *eBioMedicine*.

[7] Dai R., Wang Z., Samanipour R., Koo K. I., Kim K. (2016). Adipose-derived stem cells for tissue engineering and regenerative medicine applications. *Stem Cells International*.

[8] Lei H., Xin H., Guan R. (2015). Low-intensity Pulsed Ultrasound Improves Erectile Function in Streptozotocin- induced Type I Diabetic Rats. *Urology*.

[9] Cui W., Li H., Guan R. (2019). Efficacy and safety of novel low-intensity pulsed ultrasound (LIPUS) in treating mild to moderate erectile dysfunction: a multicenter, randomized, double-blind, sham-controlled clinical study. *Translational Andrology and Urology*.

[10] Xia S. J., Chen H. R., Li Z. (2020). Efficacy and safety of low-intensity pulsed ultrasound at different intervals by mechanical force in treating erectile dysfunction: a preliminary study. *Zhonghua Yi Xue Za Zhi*.

[11] Kang P. L., Huang H. H., Chen T., Ju K. C., Kuo S. M. (2019). Angiogenesis-promoting effect of LIPUS on hADSCs and HUVECs cultured on collagen/hyaluronan scaffolds. *Materials science & engineering C, Materials for biological applications*.

[12] Xia B., Chen G., Zou Y., Yang L., Pan J., Lv Y. (2019). Low-intensity pulsed ultrasound combination with induced pluripotent stem cells-derived neural crest stem cells and growth differentiation factor 5 promotes sciatic nerve regeneration and functional recovery. *Journal of Tissue Engineering and Regenerative Medicine*.

[13] Chen C., Zhang T., Liu F. (2019). Effect of low-intensity pulsed ultrasound after autologous adipose-derived stromal cell transplantation for bone-tendon healing in a rabbit model. *The American Journal of Sports Medicine*.

[14] Heaton J. P., Varrin S. J., Morales A. (1991). The characterization of a bio-assay of erectile function in a rat model. *The Journal of Urology*.

[15] Liu Q., Cui Y., Lin H. (2019). MicroRNA-145 engineered bone marrow-derived mesenchymal stem cells alleviated erectile dysfunction in aged rats. *Stem Cell Research & Therapy*.

[16] Love M. I., Huber W., Anders S. (2014). Moderated estimation of fold change and dispersion for RNA-seq data with DESeq2. *Genome Biology*.

[17] (2015). R K: pheatmap: pretty heatmaps. *R package version 1012*.

[18] Huang W., Sherman B. T., Lempicki R. A. (2009). Systematic and integrative analysis of large gene lists using DAVID bioinformatics resources. *Nature Protocols*.

[19] Huang W., Sherman B. T., Lempicki R. A. (2009). Bioinformatics enrichment tools: paths toward the comprehensive functional analysis of large gene lists. *Nucleic Acids Research*.

[20] Szklarczyk D., Franceschini A., Wyder S. (2015). STRING v10: protein-protein interaction networks, integrated over the tree of life. *Nucleic Acids Research*.

[21] Zhang G., Li X., Wu L., Qin Y. X. (2021). Piezo1 channel activation in response to mechanobiological acoustic radiation force in osteoblastic cells. *Bone research*.

[22] Pathak M. M., Nourse J. L., Tran T. (2014). Stretch-activated ion channel Piezo1 directs lineage choice in human neural stem cells. *Proceedings of the National Academy of Sciences of the United States of America*.

[23] Giuliano F. (2009). Mechanism of action of PDE5 inhibitors in LUTS and ED: the NO-cGMP pathway. *European Urology*.

[24] Burnett A. L., Lowenstein C. J., Bredt D. S., Chang T. S., Snyder S. H. (1992). Nitric oxide: a physiologic mediator of penile erection. *Science*.

[25] Wang J., Mi Y., Wu S. (2020). Exosomes from adipose-derived stem cells protect against high glucose-induced erectile dysfunction by delivery of corin in a streptozotocin-induced diabetic rat model. *Regenerative Therapy*.

[26] Zhang H. B., Chen F. Z., He S. H. (2019). In vivo tracking on longer retention of transplanted myocardin gene-modified adipose-derived stem cells to improve erectile dysfunction in diabetic rats. *Stem Cell Research & Therapy*.

[27] Zhou J., Yin Y., Yang Y. (2021). Knockdown of miR-423-5p simultaneously upgrades the eNOS and VEGFa pathways in ADSCs and improves erectile function in diabetic rats. *Journal of Cellular and Molecular Medicine*.

[28] Wang X., Liu C., Xu Y. (2017). Combination of mesenchymal stem cell injection with icariin for the treatment of diabetes-associated erectile dysfunction. *PLoS One*.

[29] Xu J., Liu X., Zhao F., Zhang Y., Wang Z. (2020). HIF1*α* overexpression enhances diabetic wound closure in high glucose and low oxygen conditions by promoting adipose-derived stem cell paracrine function and survival. *Stem Cell Research & Therapy*.

[30] Chen X., Yan L., Guo Z. (2016). Adipose-derived mesenchymal stem cells promote the survival of fat grafts via crosstalk between the Nrf2 and TLR4 pathways. *Cell Death & Disease*.

[31] Tan Y., Guo Y., Reed-Maldonado A. B. (2021). Low-intensity pulsed ultrasound stimulates proliferation of stem/progenitor cells: what we need to know to translate basic science research into clinical applications. *Asian Journal of Andrology*.

[32] Costa V., Carina V., Fontana S. (2018). Osteogenic commitment and differentiation of human mesenchymal stem cells by low-intensity pulsed ultrasound stimulation. *Journal of Cellular Physiology*.

[33] Ren C., Chen X., Du N. (2018). Low-intensity pulsed ultrasound promotes Schwann cell viability and proliferation via the GSK-3*β*/*β*-catenin signaling pathway. *International Journal of Biological Sciences*.

[34] Ling L., Wei T., He L. (2017). Low-intensity pulsed ultrasound activates ERK1/2 and PI3K-Akt signalling pathways and promotes the proliferation of human amnion-derived mesenchymal stem cells. *Cell Proliferation*.

[35] Cores J., Hensley M. T., Kinlaw K. (2017). Safety and efficacy of allogeneic lung spheroid cells in a mismatched rat model of pulmonary fibrosis. *Stem Cells Translational Medicine*.

[36] Zlabinger K., Lukovic D., Hemetsberger R. (2018). Matrix metalloproteinase-2 impairs homing of intracoronary delivered mesenchymal stem cells in a porcine reperfused myocardial infarction: comparison with Intramyocardial cell delivery. *Frontiers in Bioengineering and Biotechnology*.

[37] Albersen M., Berkers J., Dekoninck P. (2013). Expression of a distinct set of chemokine receptors in adipose tissue-derived stem cells is responsible for in vitro migration toward chemokines appearing in the major pelvic ganglion following cavernous nerve injury. *Sexual Medicine*.

[38] Vanden Berg-Foels W. S. (2014). In situ tissue regeneration: chemoattractants for endogenous stem cell recruitment. *Tissue Engineering. Part B, Reviews*.

[39] Yamaguchi J., Kusano K. F., Masuo O. (2003). Stromal cell-derived factor-1 effects on ex vivo expanded endothelial progenitor cell recruitment for ischemic neovascularization. *Circulation*.

[40] Hardy D., Fefeu M., Besnard A. (2019). Defective angiogenesis in CXCL12 mutant mice impairs skeletal muscle regeneration. *Skeletal Muscle*.

[41] Simons M., Ware J. A. (2003). Therapeutic angiogenesis in cardiovascular disease. *Nature Reviews Drug Discovery*.

[42] Apte R. S., Chen D. S., Ferrara N. (2019). VEGF in signaling and disease: beyond discovery and development. *Cell*.

[43] Cross M. J., Claesson-Welsh L. (2001). FGF and VEGF function in angiogenesis: signalling pathways, biological responses and therapeutic inhibition. *Trends in Pharmacological Sciences*.

[44] Chen J., Jiang J., Wang W. (2019). Low intensity pulsed ultrasound promotes the migration of bone marrow- derived mesenchymal stem cells via activating FAK-ERK1/2 signalling pathway. *Artificial Cells, Nanomedicine, and Biotechnology*.

[45] Coste B., Mathur J., Schmidt M. (2010). Piezo1 and Piezo2 are essential components of distinct mechanically activated cation channels. *Science*.

[46] Kefauver J. M., Ward A. B., Patapoutian A. (2020). Discoveries in structure and physiology of mechanically activated ion channels. *Nature*.

[47] Murthy S. E., Dubin A. E., Patapoutian A. (2017). Piezos thrive under pressure: mechanically activated ion channels in health and disease. *Nature Reviews Molecular Cell Biology*.

[48] Shujie X., Yanjie G. (2021). Progress on the activation of endogenous stem cells and functional repair in LIPUS mechanotransduction. *Journal of Shandong University*.

[49] Menghao S., Shujie X. (2020). Research progress on the activation of endogenous stem cells in the mechano-biological chain for the treatment of erectile dysfunction. *Chinese Journal of Medicine*.

[50] Moroni M., Servin-Vences M. R., Fleischer R., Sánchez-Carranza O., Lewin G. R. (2018). Voltage gating of mechanosensitive PIEZO channels. *Nature Communications*.

[51] Lavoie H., Gagnon J., Therrien M. (2020). ERK signalling: a master regulator of cell behaviour, life and fate. *Nature Reviews Molecular Cell Biology*.

[52] Holmes D. I., Zachary I. (2005). The vascular endothelial growth factor (VEGF) family: angiogenic factors in health and disease. *Genome Biology*.

