# Supplementary material for: Low-Intensity Pulsed Ultrasound Enhanced Adipose-Derived Stem Cell-Mediated Angiogenesis in the Treatment of Diabetic Erectile Dysfunction through the Piezo-ERK-VEGF Axis
Source: Stem Cells Int. 2022 Jul 29;2022:6202842. doi: 10.1155/2022/6202842 (PMC9355763; doi:10.1155/2022/6202842)
Supplement: Supplementary Materials — Table 1: Primers used for qRT–PCR. Figure 1 The fate of transplanted ADSCs. No significant differences in the number of resident stem cells were observed one day after the transplantation. 3 and 5 days later, more ADSCs were retained in the cavernosum in the LIPUS treatment group. Data are presented as the mean ± standard deviation. ns p > 0.05 when comparing the ADSC group on the first day. ∗p < 0.05 when comparing the ADSC group on the third and fifth days. Figure 2 The EdU staining of ADSCs. The percentage of EdU-positive cells in the LIPUS treatment group was significantly higher than that in the NC group. Data are presented as the mean ± standard deviation. ∗p < 0.05 when comparing the NC group. Figure 3 The protein–protein interaction network of DEGs of ADSCs without and with LIPUS stimulation. [file 6202842.f1.pdf]

Primer	Primer Sequence
CXCL12-F	TGCCGATTCTTTGAGAGCCATGTC
CXCL12-R	GCAACAATCTGAAGGGCACAGTTTG
FGF2-F	CAAGCAGAAGAGAGAGGAGTTGTGTC
FGF2-R	CAGCCGTCCATCTTCCTTCATAGC
VEGF-F	CACCAAAGCCAGCACATAGGAGAG
VEGF-R	GTCTGCGGATCTTGGACAAACAAATG
NGF-F	GGCGAGGTGAACATTAACAACAGTG
NGF-R	GTGTGAGTCGTGGTGCAGTATGAG
HGF-F	TCAGGACCTTGTGAGGGAGATTATGG
HGF-R	ACCAGGAACAATGACACCAAGAACC
IGF1-F	GAGATGTACTGTGCTCCGCTGAAG
IGF1-R	AGTGTACTTCCTTTCCTTCTCCTTTGC
GAPDH-F	CCACCAACTGCTTAGCCCCC
GAPDH-R	GCAGTGATGGCATGGACTGTGG

Supplementary Table 1 Primers used for qRT–PCR.

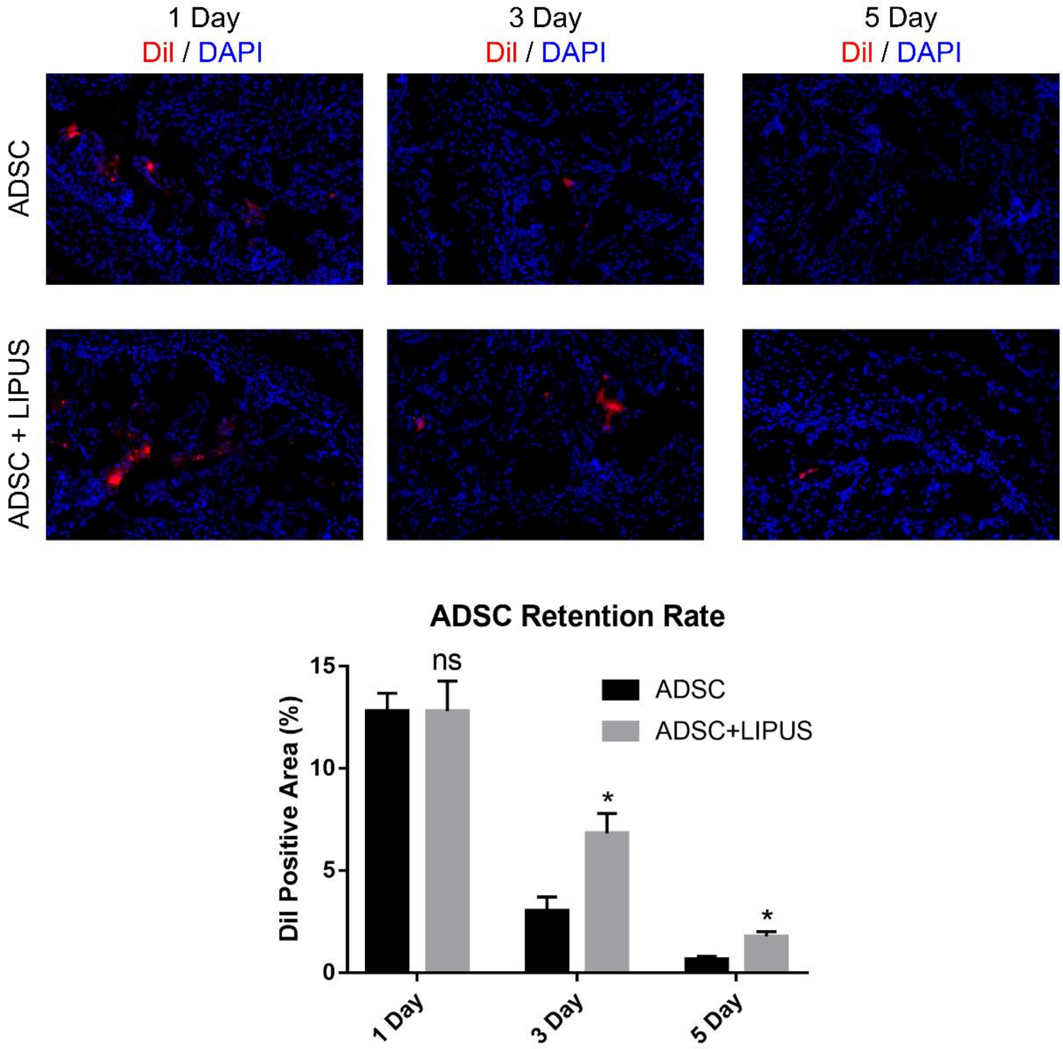

Supplementary Figure 1 The fate of transplanted ADSCs. No significant differences in the number of resident stem cells were observed one day after the transplantation. 3 and 5 days later, more ADSCs were retained in the cavernosum in the LIPUS treatment group. Data are presented as the mean ± standard deviation. ns $p > 0.05$ when comparing the ADSC group on the first day. * $p < 0.05$ when comparing the ADSC group on the third and fifth days.

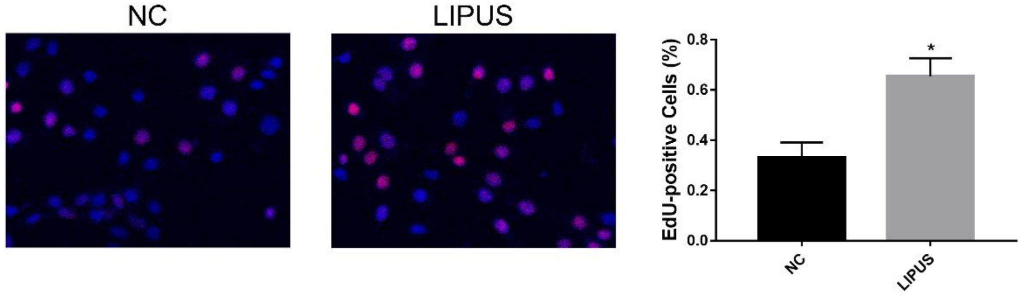

Supplementary Figure 2 The EdU staining of ADSCs. The percentage of EdU-positive cells in the LIPUS treatment group was significantly higher than that in the NC group. Data are presented as the mean ± standard deviation. * $p < 0.05$ when comparing the NC group.

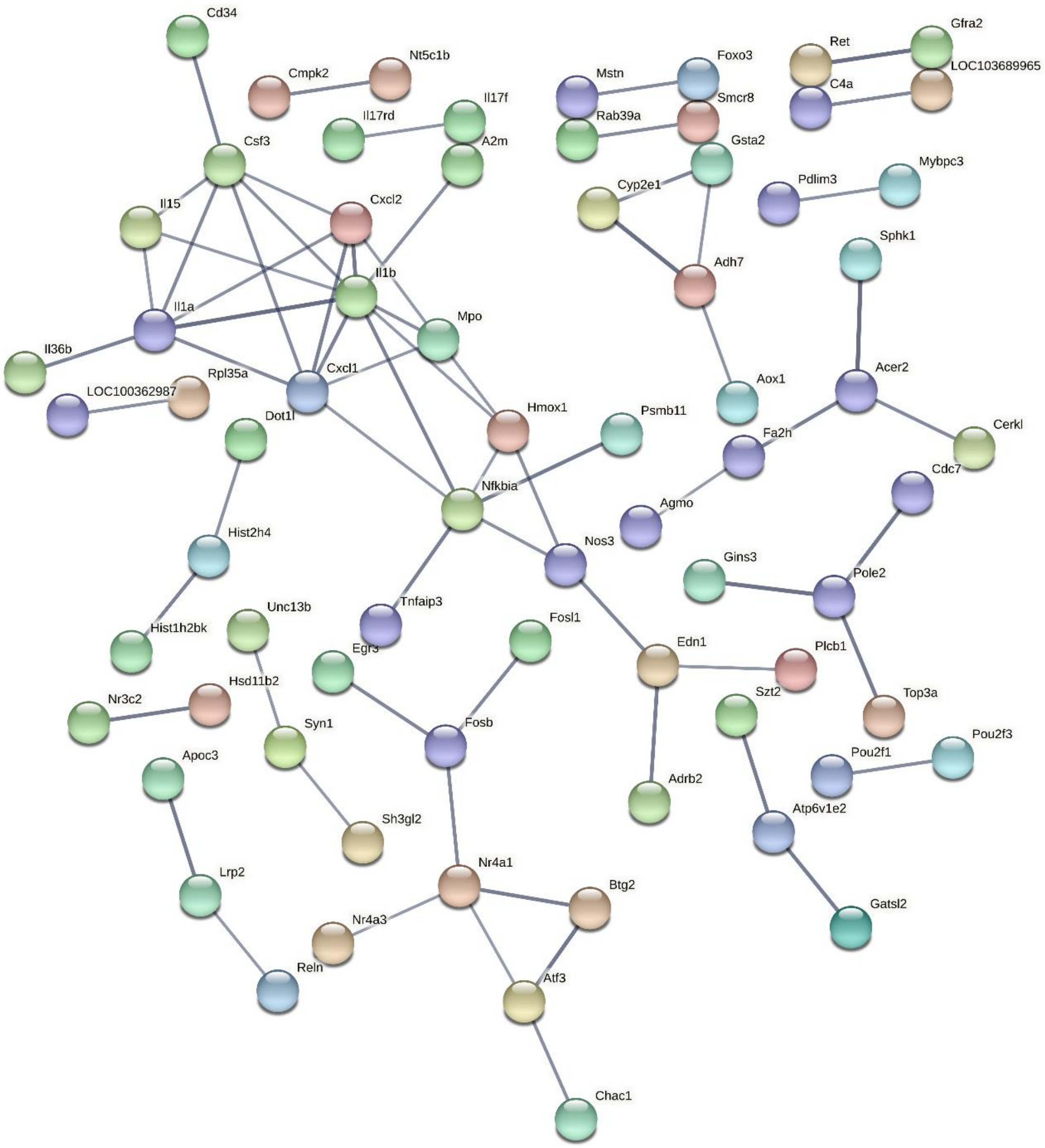

Supplementary Figure 3 The protein–protein interaction network of DEGs of ADSCs without and with LIPUS stimulation.
